# Scientific Evidence and Common Perceptions of Factors Affecting Sugar Content in Pasture Grass: Is There a Link With Pre‐existing Horse‐Related Experience?

**DOI:** 10.1002/vms3.70778

**Published:** 2026-01-18

**Authors:** Isabel Moaby, Alex Aitken, Sandra Varga

**Affiliations:** ^1^ School of Natural Sciences University of Lincoln Lincoln UK

**Keywords:** equine nutrition, fungi, laminitis, management, sugar content

## Abstract

**Background:**

Several equine conditions are associated with and exacerbated by increased high‐sugar grass intake. Knowing how climatic and biotic factors affect sugar content in grasses is important for decision‐making by those involved in the management of equines.

**Objectives:**

(1) To characterise equine owners’ knowledge and perceptions of the factors affecting sugar content in grasses to inform in the management of grasses and equines. (2) To identify associations between pre‐existing horse‐related experience and level of knowledge about equine nutrition and health conditions.

**Methods:**

A questionnaire was developed and distributed online to characterise the perceptions of those involved in the management of equines and their knowledge of the environmental factors known to impact grass non‐structural carbohydrate (NSC) levels, describing also the extent to which these factors associated with participants’ level of experience in equine management.

**Results:**

194 self‐declared equine owners or responsible for equines completed the survey. Our results indicate that participants were relatively well informed regarding only some of the environmental factors known to affect sugar content in grasses, and less so in relation to how the presence of fungi, overgrazing/rotational stocking might influence NSC, indicating a significant gap in knowledge. The level of previous experience with equines was not associated with more accurate knowledge, highlighting the need for facilitating more knowledge exchange activities between stakeholders and the scientific community.

**Conclusions:**

We suggest that enhancing the dissemination of the effects of plant‐fungal interactions and rotational stocking on NSC within the equine community may further improve their understanding around NSC content in grasses and its management, as fungi could be used to manage grass establishment and growth in paddocks and the grass sugar content.

## Graphical abstract

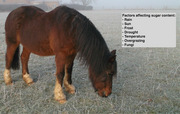

## 1 Introduction

In horse diets, grass can constitute between 50% and 100% of the total diet in a dry matter basis and provides the nutrients and non‐structural carbohydrates (NSC) that the animals need to function correctly (Virkajärvi et al. 2012). There are several diseases of horses known to be associated and exacerbated by high/increased high‐sugar grass intake (reviewed by Secombe and Lester 2012). For example, laminitis is one of the most common diseases of horses, naturally affecting between 1.5% and 34% of them depending on the study (Wylie et al. 2011). Laminitis is a painful and debilitating condition of the hoof that has major impacts on the welfare of the animal and can lead to euthanasia (Hopster and van Eps 2019). Laminitis has a complex multifactorial origin not yet completely understood. Several diet‐related factors are suggested to predispose to laminitis, including a high sugar intake due to the NSC overload (Pollitt et al. 2003; van Eps and Pollitt 2006).

Plants use CO_2_ from the atmosphere to obtain carbon and use most of it for their metabolism (i.e., respiration) and to build their biomass (cellulose, lignin). The rest of this carbon is in the form of NSC, which include soluble sugars such as glucose, fructose and sucrose, starch and fructans (Jensen et al. 2014). NSC support plant metabolism at night or when photosynthesis is not sufficient to meet the plant's demands (Smith and Stitt 2007). The dynamics of NSC are complex and usually interpreted based on the idea that once the plant's current carbon energy requirements are covered by current and recent photosynthates, any excess carbon production accumulates within the non‐reproductive tissues such as leaves and roots (Barbehenn et al. 2004; Martínez‐Vilalta et al. 2016). The main storage compounds used differ between plants and significant differences are observed between cool‐season grasses (or C_3_ grasses) and warm‐season (or C_4_) grasses. Cool‐season grasses use mainly fructans as the storage compound (Miller et al. 2001). During cool weather, these grasses can accumulate large amounts of fructans mainly in cell vacuoles of the stems (Gallagher et al. 2007) that they can convert to simple sugars when needed, for example under hot or dry conditions when they are not able to photosynthesise. C_4_ grasses on the other hand, are adapted to hot, dry weather and generally accumulate starch within chloroplasts on the leaves which is not transposable within the plant and they go dormant after frost (Moore and Hatfield 1994). Because of these physiological differences, C_3_ grasses generally have higher NSC than C_4_ (Chatterton et al. 1989; DeBoer et al. 2017).

The environmental factors affecting NSC content in grasses are relatively well established in the scientific literature (Watts and Chatterton 2004) and include temperature (Chatterton et al. 1989; Espevig et al. 2011), light intensity (Comont et al. 2013), and soil water (Rogers et al. 2019) and nutrient content (Conaghan et al. 2012). Therefore, the amount of NSC in grasses may vary daily (being highest in late afternoon compared to the morning (Pelletier et al. 2009; Pelletier et al. 2010; Kagan et al. 2011; Morin et al. 2012), seasonally (being highest during early spring and autumn [Kagan et al. 2011; Jensen et al. 2014]), and even between grass cultivars (Jensen et al. 2014). However, common perceptions on the factors affecting sugar content in grasses may not always align with the available scientific evidence. Therefore, this study aimed to characterise equine owners’ knowledge and perceptions of the factors affecting sugar content in grasses to identify gaps in current knowledge and inform in the management of grasses and equines. Because horse owner's experience and level of knowledge about equine nutrition and health conditions may affect how equines are managed (Gerber et al. 2011), we also aimed to identify associations between knowledge with the level of experience with equines.

## 2 Materials and Methods

### 2.1 Ethical Approval

This research study received a favourable ethical opinion by the University of Lincoln Research Ethics Committee (Reference: UoL2022_10485). All participants were fully briefed on the study and provided informed consent. Participation was anonymous and participants could withdraw from the study at any time.

### 2.2 Methods

A cross‐sectional study design was used to develop a questionnaire using JISC online surveys software (JISC, 2022) designed to take around 20 min. The survey included questions on demographics, management of equines and paddocks, and knowledge on nine environmental factors known to affect sugar content in grasses (Table 1, and Table S1 for the full questionnaire).

**TABLE 1:** Survey items.

Topic	Question (Answer)
Demographics	Where are you located? *Open‐ended*.
	How many equines do you currently own/loan? *Open‐ended*.
	In total, how many equines have you owned/loaned? *Open‐ended*.
Management	Have you ever tested your paddock grass for sugar? *Yes / No*.
	Do your horses have a grass susceptible/grass intolerant condition? This could include issues with weight gain. *Yes / No. If Yes, please state which conditions*.
	Do you currently know what grass species are in your grazing paddocks? *Yes / No. If Yes, please state*.
Knowledge ***(correct answers bolded)***	Of these changes in environmental conditions, tick all that you think will increase sugar grass levels: ***rain*** */* ***sun*** */* ***frost*** */* ***drought*** */* ***decreased temperature*** */* ***increased temperature*** */* ***overgrazed/stressed grass*** */* ***regularly rotated grass pasture*** */* ***fungi presence***.
	Do you think that frost would increase or decrease grass sugar levels? ***Increase*** */* ***Decrease*** */ I don't know*.
	Do you think that the presence of fungi in soil or on the plant would affect the grass sugar levels within grass? ***Yes*** */ No / I don't know*.
	What time of the day do you think the grass sugar is highest? *Morning (8am – 12pm)*, ***Afternoon (12pm – 4pm)***, ***Evening (4pm – 8 pm)*,** *Night (9pm – 12am)*.

Anyone above 18 years of age, English‐speaking, who self‐declared as an equine owner or responsible for the management of equines was eligible to participate in the survey, and no incentives were provided. The questionnaire was distributed through the authors social media accounts and emails and was live from 17 November 2022 until 1 February 2023. A total of 221 people completed the survey: 27 self‐declared as non‐equine owners/responsible for equines and were therefore excluded in the final analysis.

### 2.3 Data Analysis

All data were analysed using R v4.3.1 (R Core Team 2023). The minimum number of participants needed to detect medium size differences in the data (*N* = 117) was calculated by using ‘pwr.chis.test’ function in the ’pwr’ package (Champely 2020), with a significance level of < 0.05, power (i.e., true positive probability) > 0.8 and an effect size of *w* = 0.3.

Based on the numbers of respondents on how many equines owned/were or have been responsible for, the level of horse‐related experience was classified in four broad categories: Beginner (less than 5 equines, 18.0% of answers), Intermediate (between 6 and 10 equines, 38.1% of answers), Advanced (between 11 and 100 equines, 34.0% of answers), and Expert (more than 101 equines, 9.8% of answers). This was an arbitrary cut‐off which probably does not truly reflect someone's level of experience with equines, as it would be very challenging to estimate or quantify the total level of involvement, focus of responsibility, etc. within the equine industry. Data were analysed using Chi‐square tests used to determine differences between responses. The package ‘vdc’ was used to analyse the association between answers with the level of experience using the ‘assocstats’ function (Meyer et al. 2024). Cramer's V was calculated to estimate the strength of the association between answers and level of experience.

## 3 Results

Overall, there were 194 valid responses to the questionnaire. The respondents were mainly UK‐based (88.1%), however there were also responses from Spain, Canada, United States, Australia, New Zealand, Sweden, and The Netherlands. Participants reported to have had experience with 1 to more than 1000 equines. A large proportion of responses showed that participants have had experience managing a grass‐susceptible equine condition (89.7%), being laminitis (112 participants), equine metabolic syndrome (76 participants), and seasonal weight gain (65 participants) the 3 most commonly conditions reported. Only 25.3% of participants declared to know the grass species present in their grazing paddocks (see Table S2 for a list of the species mentioned) and only 6.2% had had their paddocks analysed to measure sugar content.

### 3.1 Knowledge on Factors Increasing Sugar Content in Grass

**FIGURE 1:**
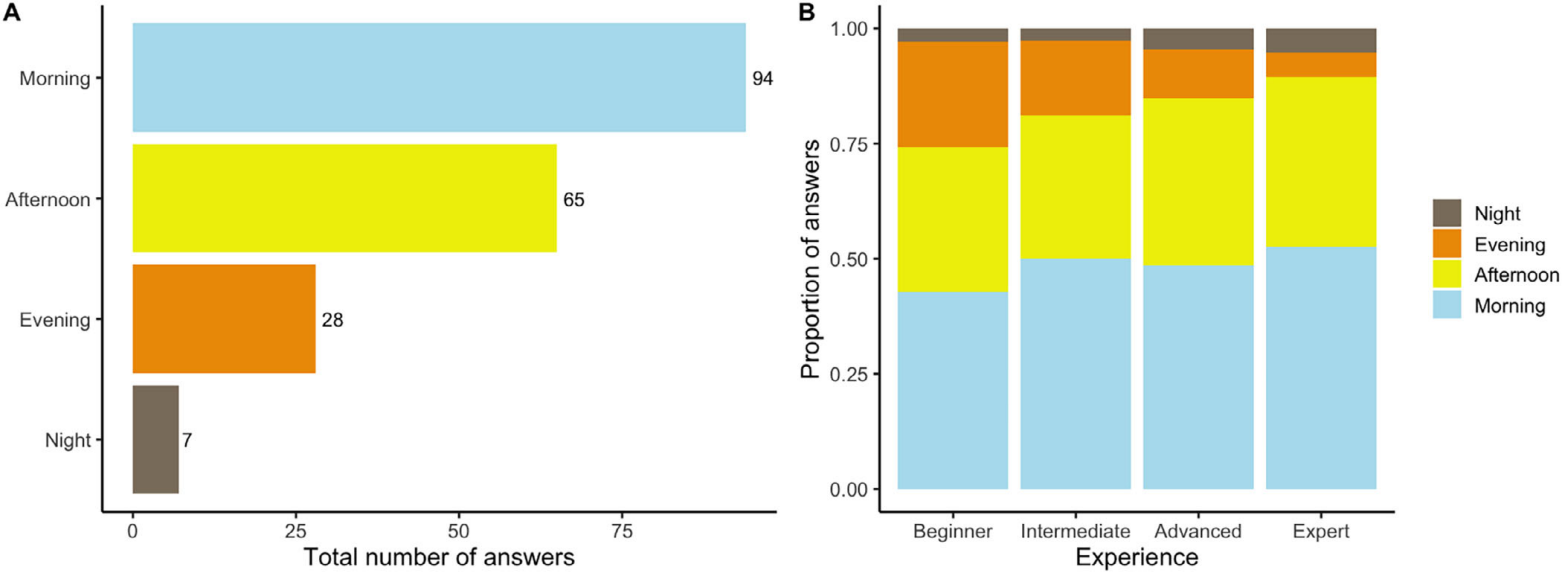
(A) Total number of answers received to the question “What time of day do you think the grass sugar is highest?” (B) Proportion of answers received by each time of the day depending on the level of Experience. According to the scientific evidence, late afternoon is when the highest level is measured, so afternoon and evening were both correct answers.

When asked about what time of the day the sugar content was highest, almost half of the participants (48%) answered during the morning, followed by afternoon (34%), evening (14%) and night (4%; Figure 1A). No significant association between the answers received and the level of equine experience was detected (*X* ^2^_9_ = 4.85, *P* = 0.84; Figure 1B).

**FIGURE 2:**
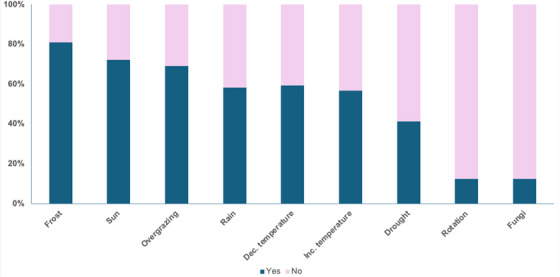
(A) Proportion of participants correctly identifying (blue bars) or not (pink bars) environmental factors known to increase sugar content in grass according to the scientific evidence. Factors are shown in decreasing order of correctly being identified.

Participants were asked to indicate which of nine given factors they thought would increase sugar content, with the expectation that participants should identify all of them. Overall, participants selected 4 factors on average, and only 10 participants selected all the factors. Frost was the factor most well‐known (81% of participants identified it), followed by sun (72%) and overgrazing/stressed grass (60%). Opposite to this, rotational stocking and fungi were only correctly identified by 12% of participants (Figure 2). There were no significant associations between the level of experience with any of the answers (Table 2).

**TABLE 2:** Results of the relationships between identifying several factors known to affect sugar content in grasses and level of equine experience using Pearson *X* ^2^ and Cramer's V.

	df	Pearson *X* ^2^	*p*‐value	Cramer's V
Time of day	9	4.92	0.84	0.092
Frost	6	3.17	0.78	0.091
Sun	3	1.61	0.65	0.091
Overgrazing	3	2.46	0.48	0.113
Rain	3	1.20	0.75	0.079
Dec. temperature	3	6.93	0.07	0.189
Inc. temperature	3	1.88	0.59	0.099
Drought	3	1.37	0.71	0.084
Rotational stocking	3	2.09	0.55	0.104
Fungi	6	7.42	0.28	0.138

Below, we present a more detailed analysis for the top and bottom factors identified by the participants and the association with their level of expertise. The differences between the level of expertise for the rest of the factors are shown as Supporting Information (Figure S1).

### 3.2 Knowledge on the Effect of Frost on Grass Sugar Levels

**FIGURE 3:**
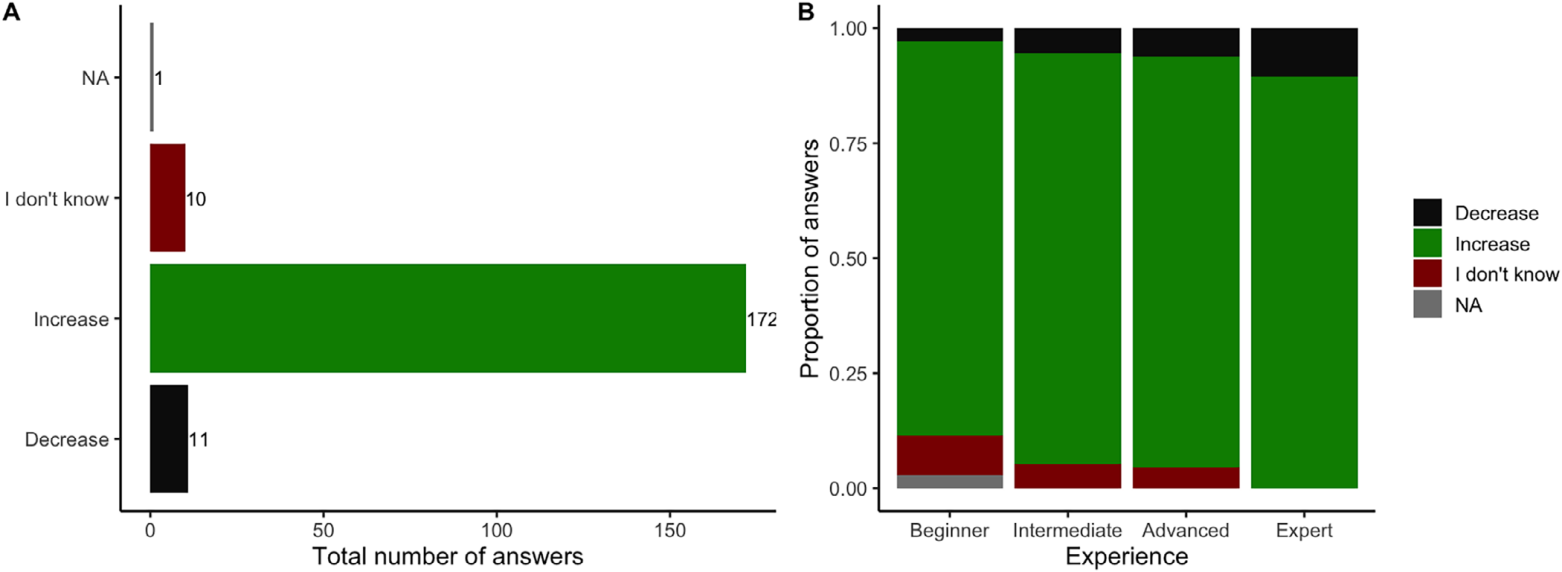
(A) Total number of answers received to the question ‘Do you think that the frost would increase or decrease grass sugar levels?’. (B) Proportion of answers received depending on the level of Experience. The effect of frost on sugar content depends on the timing of measurement after frost, and the severity and duration of the cold period. The correct answers were increase and decrease. NA indicates that an answer was not available from one participant.

Frost was correctly identified by 81% of the participants taking the survey. When asked whether frost would increase or decrease grass sugar levels, a significantly large number of participants (87%) thought that frost would increase sugar content (*X* ^2^_2_ = 270.29, *P* < 0.001; Figure 3A). There was no statistically significant association between the answers being correct with the level of experience (*X* ^2^_6_ = 3.95, *P* = 0.68), even though the number of participants that were not sure about the effect of frost on sugar content increased with decreasing level of experience (Figure 3B).

### 3.3 Knowledge on the Effect of Rotational Stocking on Grass Sugar Levels

**FIGURE 4:**
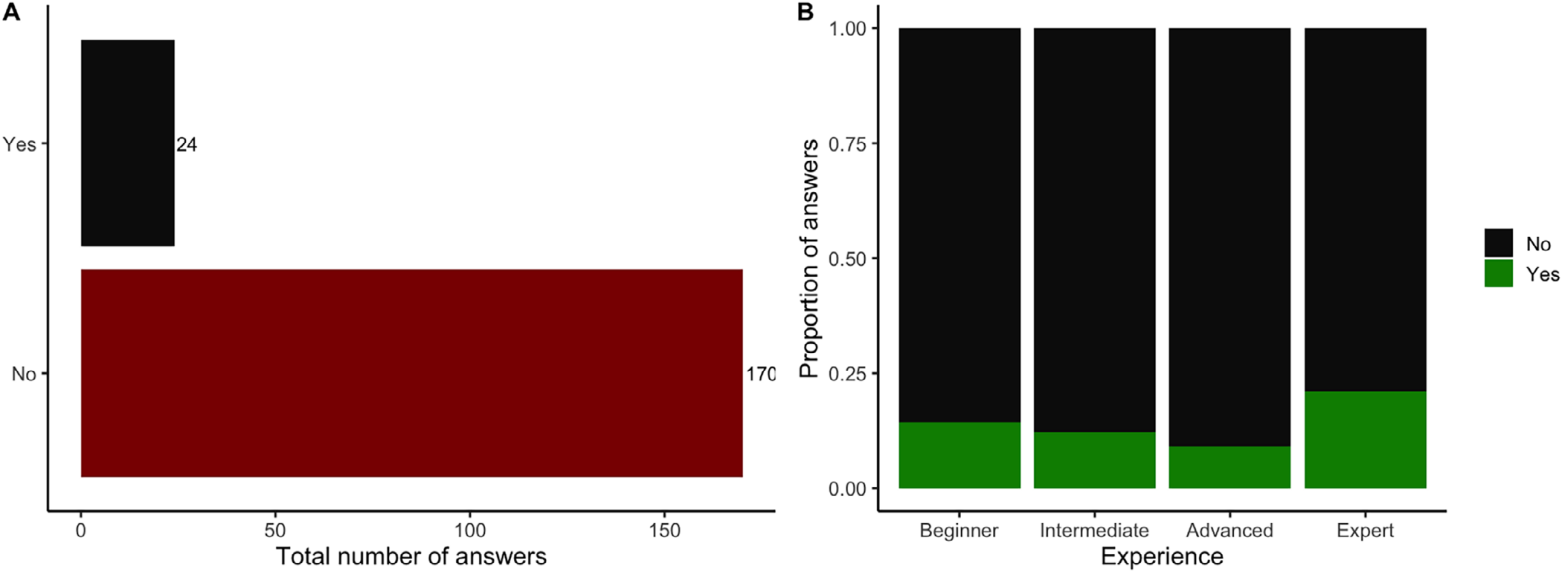
(A) Total number of answers received to the question ‘Do you think that regularly rotating grass pasture would increase grass sugar levels?’. (B) Proportion of answers received depending on the level of Experience.

The effect of rotational stocking was only identified by 12% of the participants taking the survey and most of them (87%) thought that rotational stocking would not affect sugar content (Figure 4A). There was no statistically significant association between the answers being correct with the level of experience (*X* ^2^_3_ = 2.09, *P* = 0.55), even though participants with most experience were better at correctly identifying the correct answer (Figure 4B).

### 3.4 Knowledge on the Effect of Fungi on Grass Sugar Levels

**FIGURE 5:**
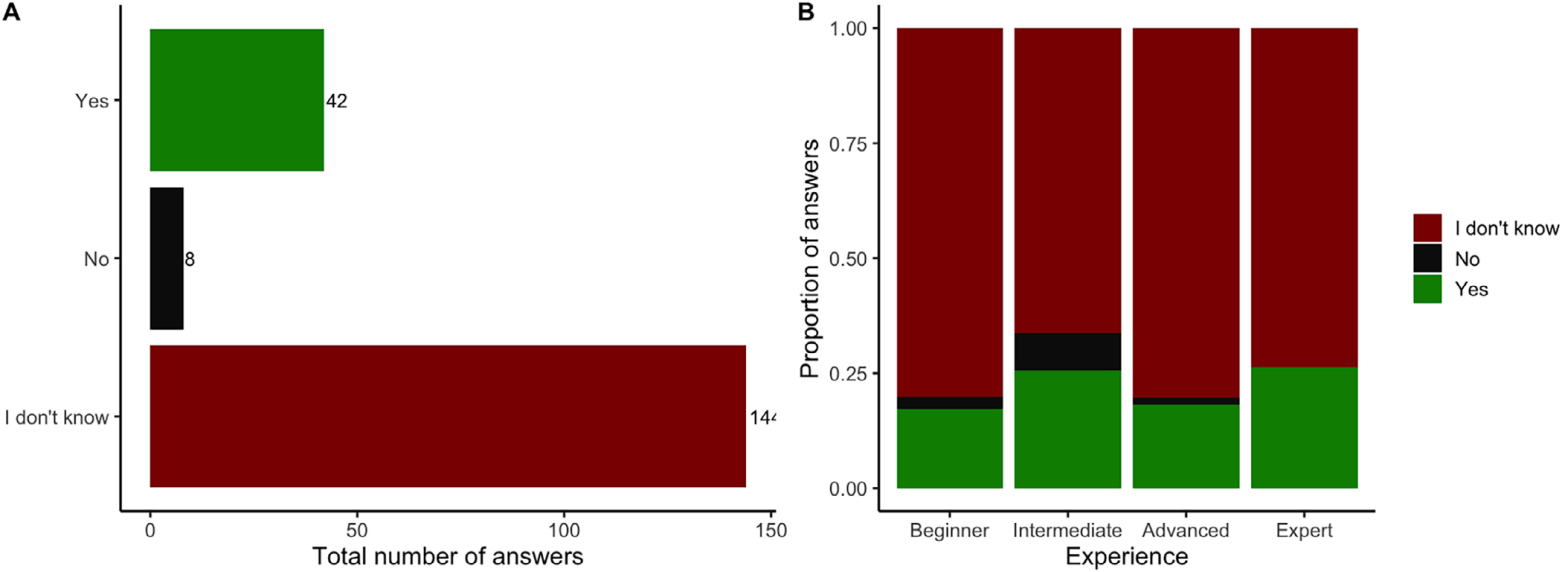
(A) Total number of answers received to the question “Do you think that the presence of fungi in soil or on the plant would affect the sugar levels within grass?”. (B) Proportion of answers received depending on the level of Experience. Depending on the fungus, NSC are expected to increase/decrease.

The majority of respondents (74%) answered that they didn't know whether the presence of fungi in soil or on the plant would affect the sugar levels within grass (*X* ^2^_2_ = 154.93, *P* < 0.001; Figure 5A). Even though there was numerical variation in the proportion of answers depending on the level of experience, no significant association was detected (*X* ^2^_6_ = 8.32, *P* = 0.621; Figure 5B).

## 4 Discussion

In this study, the perceptions of equine owners/people responsible for equines regarding the environmental factors affecting the content of sugar in grass were investigated. Our results indicate that the equine community is relatively well informed regarding some climatic factors known to affect sugar content in grasses such as frost, but much less so in relation to how the presence of fungi or rotational stocking might influence NSC. Moreover, our analysis indicates that the level of previous experience with equines is not associated with more correct knowledge about the environmental factors affecting sugar content in grasses.

The annual dynamics of NSC are well established for perennial grasses which show the highest pool of sugars in late summer or autumn in response to slow growth and leaf senescence due to seasonal drops in temperature and light (Longland and Byrd 2006; Kagan et al. 2011; Morin et al. 2012; Jensen et al. 2014; Benot et al. 2019). Daily dynamics of NSC are also well described in the scientific literature, with a peak sugar content found after midday/late afternoon during the grass growing period in spring, with concentrations tending to rise during the morning and declining overnight (Pelletier et al. 2010; Kagan et al. 2011; Morin et al. 2012; Weinert‐Nelson et al. 2022). The same patterns in increased daily concentrations of NSC are reported for non‐grasses forage species such as alfalfa (Morin et al. 2011) or red clover (Owens et al. 1999). For these reasons, morning grazing and/or restricted grazing are suggested as best practices for metabolic challenged horses (Watts 2004; EEG Equine Endocrinology Group 2024), which were expected to be known by the survey participants.

Regardless of the level of previous experience with equines, participants were relatively unaware of the effects of fungi on sugar content, which 74% of respondents indicating that they did not know what the effect could be. Grass species are usually colonised by symbiotic fungal endophytes that grow within the plant tissues either producing positive or negative effects for the plants (Sanchez Marquez et al. 2012). For example, many endophytes such as *Epichloë* spp provide grasses with increased tolerance to abiotic stresses such as drought due to the production of alkaloids and can also produce toxins which can be detrimental for herbivores consuming these grasses (Riet‐Correa et al. 2013). Moreover, grasses, like most other plants, are usually colonised by arbuscular mycorrhizal fungi in their roots. In this relationship, the mycorrhizal fungi provide water and nutrients derived from the soil to the plant in exchange of sugars, which usually translates into enhanced photosynthesis and thus growth and performance (Smith and Read 2010). These fungal associations are generally regarded as mutualistic for the host plant even though the net outcome depends on other factors such as the amount of nutrients in the soil, so the interactions range in fact from being parasitic to mutualistic (Johnson et al. 2008). In terms of sugar content, both endophytic and mycorrhizal fungi depend on the plant's carbohydrates, but also may increase plant photosynthetic capacity and the amount of NSC produced (Cheplink and Cho 2003; Hill et al. 1996; Nagabhyru et al. 2013). Thus, it can be predicted that the effect of these two types of fungi on plant's sugar content may be positive, negative, or neutral. Moreover, in terms of forage management, symbiotic fungal endophytes can be beneficial as they can provide better defence against pests, but they can also modify plant community composition by direct allelopathic effects (Franzluebbers and Hill 2005) as well as indirectly through plant‐soil feedbacks (Cripps et al. 2013), highlighting the need for more studies.

Finally, participants were also relatively unaware of the effects of rotational stocking and overgrazing on NSC. Equines commonly overgraze on preferred grasses, which positively correlates with NSC concentrations (Allen et al. 2013). Besides the potential long‐term impacts for forage composition and persistence (Martinson et al. 2015), overgrazing affects photosynthesis and carbon allocation, by changing source organs to sinks during regrowth (Martínez‐Vilalta et al. 2016; Zhang et al. 2021). It is relatively well established in the scientific literature that carbohydrates stored in the tiller bases of grasses are rapidly mobilised following grazing (Morvan‐Betrand et al. 2001), and conversion of stored NSC provides energy to support the growth of new organs (Martínez‐Vilalta et al. 2016). However, to the authors’ knowledge, research on equine pasture management is relatively scarce compared to other livestock species, highlighting the need to disseminate and consider the effects of rotational stocking for equine management (but see Weinert and Williams 2018; Williams et al. 2020).

## 5 Conclusion

According to our results, the survey's participants appeared to be relatively misinformed of the environmental factors affecting the content of NSC of the grasses they might be feeding to their equines and few of them regularly tested their paddocks. A limitation of the study is that the findings relate to self‐declared equine owners or responsible for equines, with a classification of their level of experience simply based on how many equines participants declared to have been associated with. This classification does not comprehensively include the participants’ total level of involvement, focus of responsibility, etc. within the equine industry, and thus, was arbitrary. Nevertheless, disseminating the well‐established effects of plant‐fungal interactions or information on grazing management within the equine community would be useful, as it seems to be relatively ‘invisible’ to the average owner or people responsible for an equine.

## Author Contributions

**Isabel Moaby**: conceptualisation, methodology, investigation, data curation, writing – original draft preparation. **Alex Aitken**: conceptualisation, methodology, writing – reviewing and editing. **Sandra Varga**: conceptualisation, methodology, formal analysis, visualisation, writing – reviewing and editing.

## Funding

The authors have nothing to report.

## Ethics Statement

This research study received a favourable ethical opinion by the University of Lincoln Research Ethics Committee (Reference: UoL2022_10485).

## Conflicts of Interest

The authors declare no conflicts of interest.

## Supporting information



Supplementary material: The dataset analysed in this study is available in the FigShare repository, DOI: https://doi.org/10.6084/m9.figshare.25585491.
**Supporting Table 1**: Full online questionnaire used for the study.
**Supporting Table 2**: List of the grass species (common names given by the participants with their corresponding scientific names) that the participants listed as being present in their paddocks.
**Supporting Figure 1**: Proportion of answers received depending on the level of Experience for the different factors known to increase sugar content in grass according to the scientific evidence.

## Data Availability

The dataset analysed in this study is available in the FigShare repository, https://doi.org/10.6084/m9.figshare.25585491.
