# Supplementary material for: Scientific Evidence and Common Perceptions of Factors Affecting Sugar Content in Pasture Grass: Is There a Link With Pre‐existing Horse‐Related Experience?
Source: Vet Med Sci. 2026 Jan 18;12(1):e70778. doi: 10.1002/vms3.70778 (PMC12812313; doi:10.1002/vms3.70778)

**Supporting Information**

**Table S1**. Full online questionnaire used for the study.

| **CONSENT:** | |
| --- | --- |
| 1. I confirm that I have read the information sheet on the previous page dated 17/11/22 (version 1.0), for the above study. I have had the opportunity to consider the information, ask questions and have had these answered satisfactorily. I understand that my participation is voluntary and that I am free to withdraw at any time without giving any reason, without being disadvantaged in any way. I understand that should I withdraw then the information I have given us up to this point will be deleted/destroyed. However, once the anonymised data set has been created it will not be possible to remove my anonymised data from the analysis. I understand that individuals from the University of Lincoln may look at research data collected during the study, to ensure that the study is conducted appropriately. I give permission for these individuals to have access to my research data. I understand that the information collected about me will be used to support other research in the future and may be shared anonymously with other researchers. | YES / NO |
| 2. I agree to take part in the above study. If you select yes please use the tabs at the bottom of the page to move forwards and backwards though the questions, do not use the back arrow on the top ribbon of the browser page as this takes you out of the survey. | YES / NO |
| **QUESTIONS** | |
| 3. Do you or have you ever owned/loaned an equine or consider yourself involved with or knowledgeable about equines? | YES / NO |
| 4. Where are you located? Please state country and county/district. | FREE TEXT |
| **For participants answering YES to Q3:** | |
| 5. If you have equines please tell us how many you are currently responsible for? If none please put 0. | FREE TEXT |
| 6. In total, how many equines have you previously owned/loaned/consider yourself having been involved with? If none please put 0. (How "involved" is up to you to decide, for example this could include riding lessons). | FREE TEXT |
| 7. What time of day do you think the grass sugar is highest? | 8am – 12pm  12pm – 4pm  4pm – 8 pm  8pm – 12am |
| 8. Of these changes in environmental conditions, tick all that you think will increase grass sugar levels: | Rain  Sun  Frost  Drought  Decreased temperature  Increased temperature  Overgrazed/stressed grass  Regularly rotated grass pasture  Fungi presence |
| 9. Do you think that frost would increase or decrease grass sugar levels? | INCREASE / DECREASE / DON’T KNOW |
| 10. Do you think that the presence of fungi in soil or on the plant would affect the sugar levels within grass? | INCREASE / DECREASE / DON’T KNOW |
| 11. Have you ever had your paddock tested for grass sugar? | YES / NO |
| 12. What equine conditions do you believe are associated with high grass sugar? Please state. | FREE TEXT |
| 13. Do your equines or equines you are involved with have a grass susceptible or grass intolerant condition? This could include issues with weight gain. | YES / NO |
| 14. If you replied YES to the previous question, please state which conditions. If it is not on the list please specify in OTHER. | Laminitis  Equine metabolic syndrome (EMS)  Polysaccharide storage myopathy (PSSM)  Cushing’s disease/ pituitary pars intermedia dysfunction (PPID)  Gastric ulcers  Seasonal weight gain  Ongoing weight gain  Other (Please Specify) |
| 15. Do you currently know what grass species are in your grazing paddocks? (If YES, please state species) | YES / NO |

**Table S2**. List of the grass species (common names given by the participants with their corresponding scientific names) that the participants listed as being present in their paddocks.

| **Common name given** | **Scientific names** |
| --- | --- |
| Brown bent grass | *Agrostis canina* |
| Brown top | *Agrostis capillaris* |
| Foxtail | *Alopecurus* spp |
| Sweet vernal | *Anthoxanthum odoratum* |
| Cape weed | *Arctoptheca calendula* |
| False oat | *Arrhenatherum elatius* |
| Wild oats | *Avena fatua* |
| Kikuyu | *Cenchrus clandestinus* |
| Crested dogs foot | *Cynosurus cristatus* |
| Cocksfoot/Orchard grass | *Dactylis glomerata* |
| Creeping fescue/Red fescue | *Festuca rubra* |
| Fescue | *Festuca* spp |
| Yorkshire fog | *Holcus lanatus* |
| Barley | *Hordeum vulgare* |
| Rye low endophyte | *Lolium perenne* |
| Ryegrass | *Lolium* spp |
| Crowngrass | *Paspalum* ssp |
| Timothy | *Phleum pratense* |
| Meadow grass | *Poa annua* |
| Winter annual grass | *Poa annua* |
| Rough meadow grass | *Poa trivialis* |
| Old rye | *Secale cereale* |

**Figure S1.** Proportion of answers received depending on the level of Experience for the different factors known to increase sugar content in grass according to the scientific evidence.


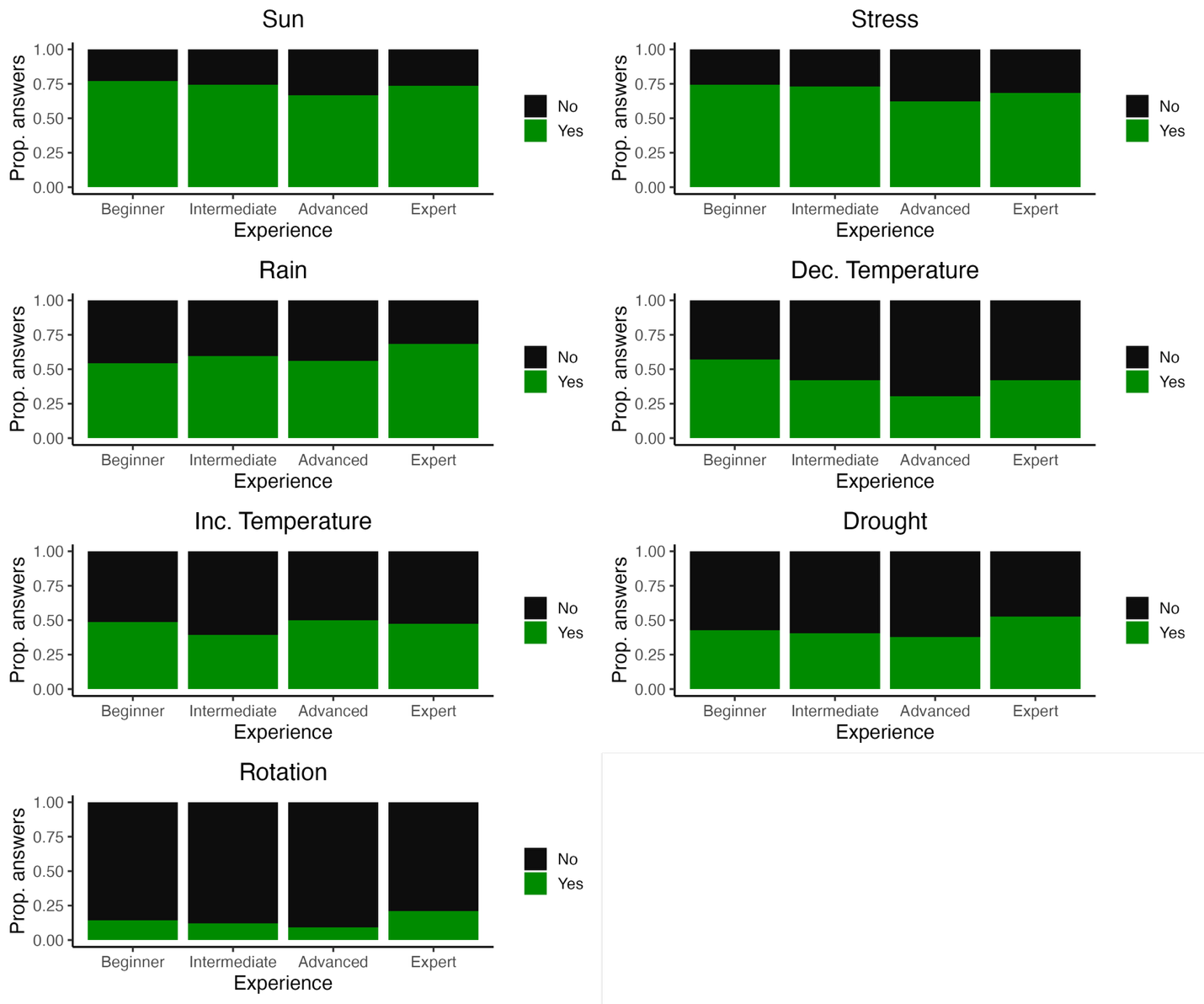

Supplement: Supplementary file 1 — Supplementary material: The dataset analysed in this study is available in the FigShare repository, DOI: https://doi.org/10.6084/m9.figshare.25585491. Supporting Table 1: Full online questionnaire used for the study. Supporting Table 2: List of the grass species (common names given by the participants with their corresponding scientific names) that the participants listed as being present in their paddocks. Supporting Figure 1: Proportion of answers received depending on the level of Experience for the different factors known to increase sugar content in grass according to the scientific evidence. [file VMS3-12-e70778-s001.docx]
